# A Case of Western Fever Treated Homœopathically — Chronic Congestion of Liver and Spleen — Neuralgia — Otitis, with Subsequent Meningitis, and Softening of the Brain

**Published:** 1850-01

**Authors:** C. D. Griswold

**Affiliations:** Batavia


ART. III.—*A Case of Western Fever treated Homœopathically—chronic congestion of Liver and Spleen—Neuralgia—Otitis, with subsequent Meningitis, and softening of the Brain.* By C. D. GRISWOLD, M. D.

On July 22d, I was called some distance from this place, to see D. T., aged 41, then suffering from severe neuralgia of the lower extremities, of some two weeks standing. The history of the case was as follows: D. T., possessing an excellent constitution, had been in the habit of making a business tour through Wisconsin and northern Illinois, during the fall of the five preceding years. While there in '43 or '44, he contracted intermittent fever, since which, he has never considered his health as perfect as before. In the fall of '48, after having suffered for several days, from exposure to the cold rains of that season, he was again attacked with fever, on arriving at Racine, Wis. From homœopathic treatment, or the "*vis medicatrix naturæ,*" or both, he recovered sufficiently in fourteen days, to pursue his journey homeward, where he arrived, still feeble, yet kept about, visited New York, but returned no better, and therefore commenced treatment with a respectable physician of this place, who, after making use of other means, resorted to salivation, with the hope of effecting an altered action, for permanent good. Mercurials acted very promptly, the third powder affecting the gums, but were continued until sixteen had been taken.

A few months after this, I first saw D. T. in this place, but not professionally, he having come here to be more particularly under the direction of his physician. His appearance was very marked. Countenance leucophlegmatic—hands shriveled and nails blue; suffering from cold at ordinary temperature. Upon his back and extremities, he informed me, were a number of small abscesses which discharged unhealthy matter. Breath extremely fœtid, and gums very much absorbed. During his stay here, he had a severe attack of otitis, which was extremely painful, and terminated in a free discharge from the external ear.

About six weeks previous to the above date, July 22d, he returned home, where I was called to prescribe for him. On examination, I found, besides the above described appearances, enlargement of the liver and spleen, and indications of congestion of the portal circulation. The complaint, however, which I was more particularly called to relieve, was described as a severe, shooting pain, which involved both lower extremities, usually but one at a time, sometimes commencing at the knee, and shooting up or down, and sometimes in the foot. The attacks were irregular, but generally commencing towards night, or in the evening, and usually continued until near morning. So severe was this pain, that the agonizing cries of the patient could be often heard by the neighbors at a distance of many rods, and the stoutest heart could hardly be induced to witness it. For sixteen nights had this patient suffered in this way, without sleep, while every conceiveable attitude would be tried for a change, after resting upon his shoulders, with his feet high upon the wall, for many minutes at a time.

My prescriptions at this time, were as follows, viz:—

℞.—Iodide of Potassium, ʒ*iss.*

Aqua pura. ℥ii.

*m.* S.—A teaspoonful to be taken three times daily.

℞.—Precip. Carb. Ferri. ℈.

S.—To be taken every third hour.

I also ordered Flemmings Sr. of Aconite to be used externally over the seat of pain, and the following mixture to be prepared.

℞.—Rad. Rumex. Aquat.
Rad. Taraxaci, *all* fresh, ℥viii.
Fol. Senna Alex., ℥i.
Aloes Soct., ʒi.
Aqua pura, Oiii.

*m.*—Digest at gentle heat, for six hours, and strain and evaporate to one-half, and add

Sulph. Ferri, ʒi.

Vin. Gallici, ℥iv.

S.—A table spoonful to be taken twice or thrice, daily, to induce full action of the bowels.

July 26th.—Patient came to this place, to continue treatment. This night, he escaped for the first time, the neuralgia, and slept, but it continued to occur again, quite frequently, for about four weeks, gradually subsiding during that time. The tr. aconite seemed to aggravate the difficulty. So did, also, Gramille's antidynous lotion, therefore they were discontinued after a few trials. After one weeks' use, the iod. of pot. was discontinued, and a comp. tr. of Calisaya bark was substituted in table spoonful doses. From the effects of the above mentioned applications, and the extreme tenderness in the region of the abscesses on the extremities, I adopted India rubber dressings in the following manner. A wet roller was loosely applied from the ankle to the knee, and over this was applied a thin sheet of India rubber, and held to its place by three narrow bands.

In this way, free moisture was kept up, which was almost invariably followed by relief from pain, and rest. After continuing the above course for about four weeks from the first, the countenance became changed from the blue aspect which had been so constant,—the abscesses were healed—the mercurial fetor of the breath was nearly gone—appetite good, and, as above stated, almost entire relief from pain. Under these circumstances, I discontinued the comp. mixture, and, at the suggestion of a medical friend, who I invited to see him with me, I substituted a preparation of sarsaparilla, mesereon, and iod. potass., but it invariably irritated his stomach, causing its contents to be rejected, consequently, I gave, in its place, a comp. decoc. of Calasaya bark, and the bitter woods with dandelion. This was well borne.

About this time he had another attack of otitis. I had left him, as usual, in the evening, but on the following morning, I found him with pulse 130 per minute; pain in the region of temp. bone, duller and more constant than had been the first attack; considerable swelling over this region; skin hot and dry; stomach irritable.

By repeatedly scarifying the part, warm fomentations, etc., the pulse was reduced to 90 per minute, the same day, and other symptoms very much mitigated. From this attack, I feared the brain would, or had already become more or less involved, and communicated the same to his family and friends. Continuing counter irritation back of the ears; pain and tenderness entirely passed away, although a chronic discharge continued from the external ear. Appetite returned,—food seemed well digested, and again he seemed recovering; commenced riding out almost daily; intellect clear, and mind more cheerful than it had been during his illness.

Sept. 12th.—Having been just seven weeks under my care, in this place, he returned home so much changed, apparently for the better, that his friends were sanguine of his recovery, and even his former medical attendant congratulated both him and me upon his apparent freedom from disease.

After his system had evidently undergone a change, I suggested the use of morphine, to induce sleep, but he stoutly objected to it, on the ground that any opiate had invariably effected him unpleasantly; relying, however, upon the change in the visceral engorgment, for a better effect, I persisted in its being taken, *pro re nata*, and found from it, the most happy effect.

Sept. 16th.—Saw him; continued improving.

Sept. 27th.—Was sent for in consequence of a sore mouth, which was thought to result from a cold. Mercurial fetor strong; salivation profuse· Prescribed iod. pot. gr. v, to be taken three times daily, in solution. Tonics of bark and bitter woods continued. Ordered counter irritation resumed back of ears, which had been discontinued. Patient remarked that he felt better than usual, that he had resumed his business correspondence.

Sept. 29th.—Was called to visit him, being worse. On arriving, learned that on the day of my last visit, he had eaten very heartily of lamb soup; he said three pints; on the following morning was taken with a chill, which was followed with fever. Stomach irritable; countenance anxious; salivation continuous. My fears were confirmed at first sight, and particularly so, when I learned that counter irritation had not been resumed as I directed. Applied blisters back of the ears, and administered pulv. ipec. and tart. emet. in small doses, as the most active means he would bear. He became comatose soon after, but not so but that he could be roused, and recognize his acquaintances. A few hours previous to death, his mind cleared up, communicated his last wishes in a deliberate manner, and a moment after taking the hand of a friend, expired on the morning of the third day after the above date.

*Inspection* twenty-four hours after death, assisted by Dr. H. Ganson, of this place. On raising the skull cap, three or four table spoonsfull of transparent fluid escaped. The ventricles were also somewhat distended. Dura-mater slightly attached to the pelvous portion of left temp. bone. Vessels of meninges engorged; also of the choroid plexus. Left lobe of cerebellum was softened over one-fourth its substance.

*Chest.*—A slight adhesion of apparently long standing, involving the posterior, inferior portion of the left lung. Slight ossification of aortic valve.

*Abdomen.*—Liver one-third larger than natural. Spleen twice its natural size. Mesenteric veins slightly engorged, and the Colon in its whole length was contracted to the size of the small intestines. Mucous membrane in some portions was of a bluish tint, and somewhat softened.

Batavia, Nov. 17, 1849.

